# The role of feedback and modulation in determining temperature resiliency in the lobster cardiac nervous system

**DOI:** 10.3389/fnins.2023.1113843

**Published:** 2023-03-09

**Authors:** Daniel J. Powell, Elizabeth Owens, Marie M. Bergsund, Maren Cooper, Peter Newstein, Emily Berner, Rania Janmohamed, Patsy S. Dickinson

**Affiliations:** ^1^Department of Biology, Bowdoin College, Brunswick, ME, United States; ^2^Program in Neuroscience, Bowdoin College, Brunswick, ME, United States

**Keywords:** myosuppressin, FMRFamide-like neuropeptide, nitric oxide, cardiac ganglion, crash temperature, L-arginine, temperature dependencies

## Abstract

Changes in ambient temperature affect all biological processes. However, these effects are process specific and often vary non-linearly. It is thus a non-trivial problem for neuronal circuits to maintain coordinated, functional output across a range of temperatures. The cardiac nervous systems in two species of decapod crustaceans, *Homarus americanus* and *Cancer borealis*, can maintain function across a wide but physiologically relevant temperature range. However, the processes that underlie temperature resilience in neuronal circuits and muscle systems are not fully understood. Here, we demonstrate that the non-isolated cardiac nervous system (i.e., the whole heart: neurons, effector organs, intrinsic feedback systems) in the American lobster, *H. americanus*, is more sensitive to warm temperatures than the isolated cardiac ganglion (CG) that controls the heartbeat. This was surprising as modulatory processes known to stabilize the output from the CG are absent when the ganglion is isolated. One source of inhibitory feedback in the intact cardiac neuromuscular system is nitric oxide (NO), which is released in response to heart contractions. We hypothesized that the greater temperature tolerance observed in the isolated CG is due to the absence of NO feedback. Here, we demonstrate that applying an NO donor to the isolated CG reduces its temperature tolerance. Similarly, we show that the NO synthase inhibitor L-nitroarginine (LNA) increases the temperature tolerance of the non-isolated nervous system. This is sufficient to explain differences in temperature tolerance between the isolated CG and the whole heart. However, in an intact lobster, the heart and CG are modulated by an array of endogenous peptides and hormones, many of which are positive regulators of the heartbeat. Many studies have demonstrated that excitatory modulators increase temperature resilience. However, this neuromuscular system is regulated by both excitatory and inhibitory peptide modulators. Perfusing SGRNFLRFamide, a FLRFamide-like peptide, through the heart increases the non-isolated nervous system’s tolerance to high temperatures. In contrast, perfusing myosuppressin, a peptide that negatively regulates the heartbeat frequency, decreases the temperature tolerance. Our data suggest that, in this nervous system, positive regulators of neural output increase temperature tolerance of the neuromuscular system, while modulators that decrease neural output decrease temperature tolerance.

## 1. Introduction

The nervous systems of all animals are subject to changes in temperature. Some mammalian and avian central nervous systems are under homeostatic regulation and are maintained within a few degrees Celsius. However, there exist examples of both mammals and birds that can withstand drastic variations in their internal body temperature, such as those that hibernate, enter torpor, or enter periods of dormancy (Ruf and Geiser, 2015; Junkins et al., 2022). While it is common for many physiological functions to arrest during periods of stasis, both the cardiac and nervous systems maintain function, albeit at a reduced capacity (Junkins et al., 2022). Many poikilotherms, animals that do not regulate their internal temperature, can maintain cardiac and neural function across a wide range of temperatures, which allows them to inhabit environments that experience daily and seasonal temperature changes (Fry, 1958; Hazel and Prosser, 1974; Macdonald, 1981; Prosser and Nelson, 1981; Zhurov and Brezina, 2005; Manning and Pelletier, 2009; Beverly et al., 2011; Robertson and Money, 2012; Soofi et al., 2014; Kushinsky et al., 2019).

Neural compensation in response to temperature change is not trivial as temperature affects all aspects of cellular function. Temperature resilience of pacemaker circuits is particularly important, as they are necessary for maintaining given behavioral states (Carpenter, 1967; Montgomery and Macdonald, 1990; Xu and Robertson, 1994; Busza et al., 2007; Reig et al., 2010; Tang et al., 2010; Robertson and Money, 2012; Rinberg et al., 2013; Kushinsky et al., 2019; Versteven et al., 2020; Powell et al., 2021; Ratliff et al., 2021). The relationship between a single biological process’s output and temperature often follows an exponential relationship (Mundim et al., 2020). For instance, changes in ion channel conductance can range from 2–20 fold in response to a 10°C temperature change (Zečević et al., 1985; Klöckner et al., 1990; Moran et al., 2004; Cao and Oertel, 2005; Fohlmeister et al., 2010; Kang et al., 2011; Robertson and Money, 2012; Tang et al., 2012; Yang and Zheng, 2014). Because each ion channel type can be differently affected by changes in temperature, predicting how temperature change will affect even a single neuron is not simple (Caplan et al., 2014; O’Leary and Marder, 2016; Alonso and Marder, 2020). These predictions are even more difficult to understand when extrapolating to neural circuits, as synaptic (Tang et al., 2010; Rinberg et al., 2013; Soofi et al., 2014; Städele et al., 2015; O’Leary and Marder, 2016; Alonso and Marder, 2020; Powell et al., 2021; Ratliff et al., 2021; Städele and Stein, 2022) and muscle physiology (Harri and Florey, 1977; Foldes et al., 1978; Rutkove, 2001; Racinais and Oksa, 2010; Thuma et al., 2013; Kushinsky et al., 2019; this paper) are also affected. Some processes of temperature compensation in the neural circuits of poikilotherms have been revealed (Harri and Florey, 1977; Foldes et al., 1978; Rutkove, 2001; Racinais and Oksa, 2010; Kushinsky et al., 2019). For example, within the stomatogastric ganglion (STG) of the Jonah crab, there are two coupled pattern generating circuits. While each circuit has different processes that enable temperature compensation, they are both able to maintain function across a similar range of temperature (∼7–30°C) (Tang et al., 2010; Tang et al., 2012; Rinberg et al., 2013; Soofi et al., 2014; Städele et al., 2015; Haddad and Marder, 2018; Powell et al., 2021; Ratliff et al., 2021; Städele and Stein, 2022). For instance, one such compensatory process includes similar increases in maximal conductance and gating rates for Na^+^ and K^+^ mediated ion channel currents that contribute to bursting (I_*h*_, I_*A*_, and synaptic currents). Because these processes have opposing impacts on neuron membrane potential, they offset one another as temperature is increased (Tang et al., 2010). It has been shown that exogenous application of neuromodulators that are normally present in the intact animal can extend the functional temperature range of these circuits (Städele et al., 2015; Haddad and Marder, 2018; Städele and Stein, 2022; this paper). Many of these modulatory peptides operate an inward-current that is both modulator dependent and voltage gated (I_*MI*_; Golowasch and Marder, 1992; Swensen and Marder, 2000). Activation of I_*MI*_ has been shown to allow neurons to maintain activity as temperature is increased by offsetting the temperature dependent increase in leak currents (Städele et al., 2015).

**FIGURE 1:**
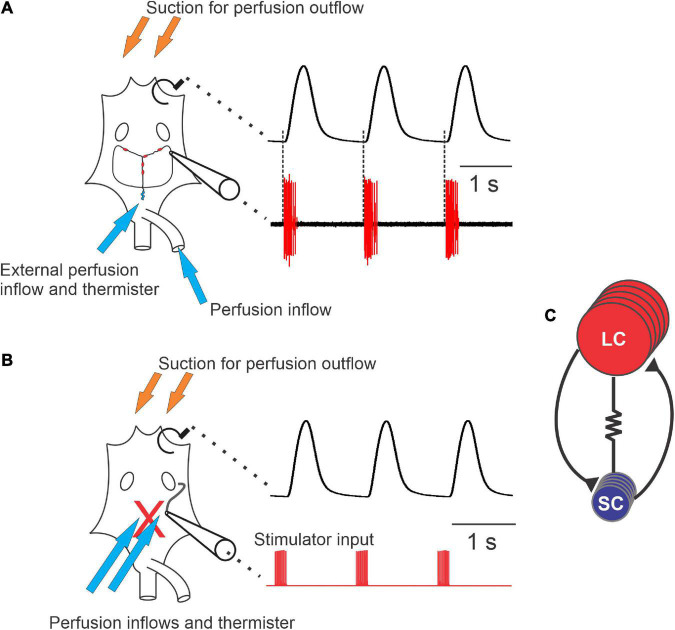
Diagram of the cardiac neuromuscular system. **(A)** A schematic of a semi-intact preparation that allowed for simultaneous recordings of the force produced from each heartbeat (upper trace) and the neuron activity of the ganglion (lower trace). The cartoon of the heart (left) shows the relative location of the cardiac ganglion, location of the small cells (SCs, small blue circles) and the locations of the large cells (LCs, red ovals). Synaptic and axonal processes are depicted as black lines connecting the neurons. Large unfilled ovals show the relative location of the ventral ostia. Note that when comparing the timing of neuron action potential bursts and the heartbeat, each burst of action potentials precedes and corresponds to a contraction of the heart (dashed lines). **(B)** Schematic of a preparation in which the neurons have been removed from heart (replaced by the red “X”), leaving only a section of nerve intact (black tortuous line connected to the cartoon electrode) that can be stimulated. The traces to the right of the schematic show that the axons left in the motor nerve can be stimulated (red, lower trace) in order to generate fictive cardiac muscle contractions (black, upper trace). **(C)** Wiring diagram of the neurons in the CG, which contains five large cells (LCs) (red) and four small cells (SCs) (blue). While all neurons are electrically coupled, this cartoon only depicts coupling between neuron types (resistor symbol). Both large and small cells excite one another *via* chemical synapses (black lines terminating in black triangles). Arrows in panels **(A,B)** show approximate locations of perfusion inflows (blue) and outflows (orange), as well as the location of the thermistors used to measure temperature.

While the cardiac nervous system in decapod crustaceans has been shown to maintain function across an ecologically relevant temperature range (Camacho et al., 2006; Worden et al., 2006; Hamilton et al., 2007; Kushinsky et al., 2019), the processes that contribute to temperature resiliency in this nervous system are not fully known. Given that the neural circuit governing the heartbeat (the cardiac ganglion, nine neurons) has substantially fewer neurons than the STG (∼30 neurons), and that the circuit is configured differently, it is unclear that the same principles will confer temperature compensation in this nervous system. To address this, we used the cardiac nervous system found in the American lobster (*H. americanus*). The lobster heartbeat is driven by a nine-neuron pacemaker circuit called the cardiac ganglion (CG). The four Small Cells (SCs) are pacemaker neurons that rhythmically, synchronously excite five Large Cells (LCs; motor neurons) that drive the cardiac muscle contractions (Figure 1). All nine neurons are electrically coupled with recurrent excitatory, chemical synaptic connections between the SCs and LCs. Consequently, the output of this circuit is a monophasic burst of action potentials that results in the synchronous muscle contractions that constitute the heartbeat (Cooke, 2002). Given that the heartbeat is responsible for circulating nutrients to all tissues in these animals as well as delivering the modulators that confer temperature robustness to the STG, we would expect that the CG is capable of maintaining stable output across a physiological range of temperatures.

The neurons of the CG and the cardiac muscles are known to be regulated by a variety of processes, including two intrinsic feedback systems. Each heart contraction activates stretch receptors that positively feed back onto the CG neurons and result in an increase in heartbeat frequency (Dickinson et al., 2016). Nitric oxide (NO) synthase, located in the cardiac muscles, releases NO, which is a negative regulator of the heartbeat (Mahadevan et al., 2004). Application of NO donors mimics this negative feedback pathway, resulting in a decrease in beat frequency and stroke volume in the intact heart (Mahadevan et al., 2004). However, the effects of NO on the muscle contractile force appear to be indirect, as NO does not alter muscle contraction parameters (Mahadevan et al., 2004). Instead, NO decreases the frequency of the synchronous LC and SC bursts by increasing the interburst interval of the action potential bursts. However, it does not affect the burst duration of the CG motor neurons (Mahadevan et al., 2004).

The heart is also modulated by a plethora of neuropeptides and hormones, including a variety of FLRFamide-like peptides, that are volume released into circulation by the pericardial organs (Ma et al., 2008; Chen et al., 2010). To our knowledge, these modulatory peptides act on the cardiac nervous system in a feedforward manner. While some of these modulators act directly on the pacemaker and motor neurons, some of these peptides modulate the neuromuscular junctions and/or regulate the muscles directly (Stevens et al., 2009; Dickinson et al., 2016). Here we investigate how both feedback and feedforward processes contribute to temperature resiliency in the cardiac neuromuscular system. Many studies have focused on how modulatory input increases the temperature tolerance of nervous systems (Städele et al., 2015; Haddad and Marder, 2018; DeMaegd and Stein, 2021; Städele and Stein, 2022). There are also a few that indicate either directly (Srithiphaphirom et al., 2019) or indirectly (Alonso and Marder, 2020) that decreasing neural excitability decreases temperature tolerance. Here we demonstrate that in the cardiac neuromuscular system, at least one feedforward modulator that increases excitability (SGRNFLRFamide; SGRN) increased the operational temperature range of the system, and that two negative regulators of excitability (NO and myosuppressin) decreased the temperature tolerance of this neuromuscular system.

While the goal of this study was to examine processes that affect the temperature tolerance of the cardiac ganglion and intact heart, responses to high temperatures have not been previously characterized in either of these structures. Therefore, some of the data detailed here describe the physiological responses we observed when this neuromuscular organ was subjected to a wide range of temperatures.

## 2. Materials and methods

### 2.1. Animals

Male and female American lobsters, *Homarus americanus*, were purchased from seafood retailers in Brunswick, ME. Lobsters were fed weekly with chopped squid or shrimp, and were housed in recirculating natural seawater tanks between 10°C and 12°C. To minimize the effects of the temperature at which lobsters had been caught, particularly summer vs. winter lobsters, most lobsters were acclimated at 10–12°C for a minimum of two weeks. In a subset of experiments described below (Section “2.9. Neuropeptide applications”), animals were used after shorter acclimation periods due to availability. Because these studies involved only crustaceans, the Bowdoin College Institutional Care and Use Committee (IACUC) did not require ethics approval for this study.

### 2.2. Whole heart preparations

Lobster hearts were dissected, and the activity of the cardiac neuromuscular system was recorded using techniques described previously (Stevens et al., 2009; Dickinson et al., 2018). Lobsters were anaesthetized on ice for 30–60 min before dissection. The heart was removed, still attached to the overlaying section of the dorsal thoracic carapace. It was then pinned ventral-side-up in a Sylgard 170-coated dish (Dow Corning, Midland, MI, USA) filled with cold lobster physiological saline (composition in mM: 479.12 NaCl, 12.74 KCl, 13.67 CaCl_2_, 20.00 MgSO_4_, 3.91 Na_2_SO_4_, 11.45 Trizma base, and 4.82 maleic acid; pH 7.45 at 25°C). The heart remained attached to the carapace to maintain the natural stretch present in the intact animal.

The heart was cannulated with a short (<1 cm) piece of tubing through the posterior artery (arrow in Figure 1A) and continuously perfused with cold (8–10°C) physiological saline at a flow rate of 2.5 ml/min. Saline thus entered through the artery and exited through the ostia toward the anterior end of the heart. A second perfusion line, also at a flow rate of 2.5 ml/min, superfused cold saline across the top of the heart to maintain the temperature on the exterior of the heart. A temperature probe (TA-29, Warner Instruments, Hamden, CT, USA) was fitted alongside the bottom of the external perfusion tubing, which was identical to the perfusion tubing cannulating the heart, and lay directly on the exterior ventral wall of the heart. The temperature was continuously monitored and controlled using an in-line temperature control system (CL-100 bipolar temperature controller and SC-20 solution heater/cooler; Warner Instruments). Both perfusion tubes came from the same heater/cooler, and so the temperature of the saline at the point measured by the external temperature probe was identical to that of the saline entering the heart. Hearts were maintained at a baseline temperature of 8–10°C until temperature ramps were applied.

To record heart contractions, the anterior arteries were tied with 6/0 Suture Silk to a Grass FT03 force-displacement transducer (Astro-Med, West Warwick, RI, USA) at an angle of approximately 30–45° from the horizontal plane. The anterior arteries were stretched to produce a baseline tonus of 2 g, which mimics the stretch in the intact animal. The contraction output was amplified using an ETH-250 Bridge amplifier (CB Sciences, Dover, NH, USA) with a high pass filter (4 Hz), and further amplified using a Brownlee 410 amplifier (Brownlee Precision, San Jose, CA, USA).

### 2.3. Semi-intact heart preparations

For semi-intact heart recordings, preparations were identical to whole heart preparations, with one exception: a small hole was cut into the ventral heart wall slightly posterior to the ostia. This exposed the cardiac ganglion without decreasing contraction force. A small portion of one of the anterolateral nerves was sucked into a suction electrode to record the extracellular electrical output of the ganglion while still connected to the heart muscle (Figure 1A). The electrode signal was recorded and amplified with a Model 1700 A-M Systems Differential AC Amplifier (Sequim, WA, USA) and a Model 410 Brownlee Precision Instrumentation Amplifier (Brownlee Precision, San Jose, CA, USA).

Both whole hearts and semi-intact hearts were allowed to equilibrate for one hour at control temperature (∼8–10°C) before testing.

### 2.4. Isolated cardiac ganglion

For isolated CG experiments, the cardiac ganglion was isolated from hearts that had previously been recorded as either whole heart or semi-intact preparations, or from hearts freshly dissected from lobsters. The heart was separated from the dorsal carapace and pinned to a Sylgard 170-lined dish filled with cold physiological saline. The ventral wall of the heart was opened, and the cardiac ganglion was dissected from the dorsal heart wall. For CGs that were dissected from hearts previously used in semi-intact recordings, the ganglion was removed immediately after the completion of the semi-intact recordings, with the heart and CG maintained in cold saline throughout the remainder of the dissection. The ganglion was pinned to a Sylgard 184-lined dish and superfused with cold (8–10°C) physiological saline at a rate of approximately 5 ml/min throughout the experiment.

To provide maximal temperature control and accuracy of temperature measurements at the ganglion, we used two perfusion inflows, one directed at the SC area and one at the LC region. Inflows were placed close to the ganglion (within 1 cm), and the thermistor (TA-29) used to measure temperature was placed within ∼1 mm of the ganglion, near one of the saline inflows. Outflows were placed on the far side of the ganglion, some distance (2–3 cm) away, so that the flow was directed across the ganglion. Because the CG is a distributed ganglion, not all regions of the ganglion were equally close to the inflow, so some variation in temperature among different regions of the ganglion was inevitable.

Neural activity was recorded using either stainless steel pin electrodes or a suction electrode. For the former method, a petroleum jelly well was made around one of the anterolateral nerves of the ganglion. One pin electrode was put into the well to record the electrical activity of the motor neuron axons in the anterolateral nerve; the other was placed nearby in the bath. For suction electrode recordings, a glass suction electrode was attached to one of the anterolateral nerves with a watertight seal. Signals from the pin electrode or the suction electrode were amplified using the same instrumentation as for the semi-intact preparation.

### 2.5. Stimulated heart preparation

To examine the effects of temperature on the neuromuscular junction and the cardiac muscle itself, we used a stimulated heart preparation (Stevens et al., 2009) in hearts that had previously been tested in the semi-intact configuration. After the semi-intact temperature ramp was completed, the opening in the ventral wall of the heart was extended from the dorsal abdominal artery to the ostia, leaving the anterior half of the heart intact. The ganglion was removed, leaving one anterolateral nerve long enough to be visible and accessible for stimulation. The severed nerve ending was stimulated using a suction electrode (Figure 1B). Stimuli, 0.5 ms in duration, were delivered in bursts having a frequency of 60 Hz and a burst duration of 300 ms. These bursts were repeated with a period of 1.3 s in trains of 15 bursts each. After each train of bursts, the nerve was left unstimulated for 60 s, after which another train of 15 bursts was delivered. Previous studies have found that this pattern of stimulation helps to prevent the degradation of muscle activity that occurs with continual stimulation (Stevens et al., 2009). Electrical impulses were generated using a CED Micro 1401 data acquisition board (Cambridge Electronic Design, Cambridge, UK) and controlled by a custom Spike2 (CED) sequencer file. Current was delivered using a Model 1700 A-M Systems Differential AC Amplifier (Sequim, WA, USA). Both saline perfusion tubes were set to flow into the intact portion of the heart, over the muscle and nerve ending being stimulated. Muscle contractions and injected current were recorded using the same instrumentation and software as for the semi-intact heart preparation. Sequencer files can be made available upon request.

### 2.6. Physiological recordings

All recordings (muscle force, electrical activity, stimulation) were made using CED 1401 data acquisition boards and Spike2 software (v 6,7, or 9; CED, Cambridge, UK). Data were recorded onto a Dell PC (Austin, TX, USA).

### 2.7. Temperature manipulations

Baseline functioning was recorded for 10 min at 8–10°C. The temperature of the perfused physiological saline was then increased by steps, controlled by the CL-100 temperature controller (set on fast cycle frequency) using a sequencer script in the Spike2 program. Steps were set to change instantaneously, and then remain steady for the remainder of the 1-min step time. Because the temperature of the SC-20 cannot change instantly, the change was more gradual, asymptoting at the new temperature in approximately 20 s. Temperature was increased in steps of 0.75°C, 1°C, or 1.25°C until the heart crashed. A crash was characterized as the muscles failing to beat or the ganglion failing to burst more than twice in 30 s. After the heart crashed, the temperature was decreased back to baseline at a rate of 1–2°C/min in most experiments; in some experiments, temperature was returned to baseline at 5°C/min. The heart was then allowed to stabilize at 8–10°C for at least 20 min to ensure its functioning returned to baseline. Preparations in which any crash temperatures were over 45°C were excluded. If the heart, nervous system, or muscle failed to produce physiological output when returned to baseline temperature, the crash was considered lethal, and the lobster was excluded.

For the stimulated preparation, the temperature was increased by 1.5°C (stepwise) after each set of 15 stimulation trains, until the temperature reached the crash temperature of the semi-intact preparation from which it was derived. It was maintained at the new temperature for the 60 s before the next train of stimulations was delivered, as well as during those 15 stimulus trains (i.e., another 15 s). Preliminary experiments showed that if the stimulated muscle was allowed to reach a temperature at which muscle contractions stopped entirely, activity could not be recovered upon return of temperature to baseline values. Therefore, the maximum temperature for each stimulated preparation was based on the crash temperature of that heart recorded in the semi-intact configuration.

### 2.8. Nitric oxide experimental manipulations

To determine whether nitric oxide (NO) released from the cardiac muscle (Mahadevan et al., 2004) was at least partly responsible for differences in the responses of the whole or semi-intact heart vs. the isolated CG, we conducted two types of experiments. In the first, we examined the effects of NO on the whole heart preparation or on the isolated CG. For whole heart experiments, we used the NO donor PAPA NONOate (Cayman Chemical Company, Ann Arbor, MI, USA) at a concentration of 10^–5^M (Cheng et al., 2011). This led to a clear decrease in contraction frequency and amplitude that lasted throughout the duration of the donor application. For experiments examining the effects of NO on the isolated CG, the nitric oxide donor SNAP (Cayman Chemical Company) was applied at a concentration of 10^–5^ M (Mahadevan et al., 2004) during temperature ramps. After control temperature ramps in saline, 10^–5^ M SNAP or PAPA NONOate was applied to isolated CG or whole heart preparations, respectively, through the perfusion system. After beginning the perfusion of NO donors, baseline functioning was recorded for 10 min at 8–10°C, then a temperature ramp was applied; the NO donor application was continued throughout the entirety of the temperature ramp.

In the second set of experiments, we blocked the production of NO in the whole heart using L-nitroarginine (LNA; Cayman Chemical Company), a competitive inhibitor of nitric oxide synthase (NOS). Using the whole heart preparation, the crash temperature of the heart was determined in control saline. The heart was then perfused with 3.30 × 10^–4^ M LNA at the baseline temperature for 20 min to block nitric oxide feedback before being subjected to a temperature ramp in LNA. To ensure that the NO precursor arginine did not alter the temperature resilience of the cardiac neuromuscular system, saline containing 1.1 × 10^–4^ M arginine was perfused through the heart. Crash temperature was not affected by arginine alone (data not shown).

### 2.9. Neuropeptide applications

To ask whether neuropeptides are able to stabilize the heart and increase its resiliency to temperature stress, as has been seen in other systems, we examined the effects of two native *Homarus* neuropeptides, SGRNFLRFamide (SGRN), and myosuppressin (pQDLDHVFLRFamide). Both peptides were custom synthesized by GenScript (Piscataway, NJ, USA). SGRN was dissolved in deionized water and stored as a 10^–3^ M stock solution at −20°C. Myosuppressin was initially dissolved in dimethyl sulfoxide (DMSO). The dissolved peptide was then diluted with deionized water to a solution containing 10^–3^ M myosuppressin and 10% DMSO. Previous experiments have shown that DMSO, when diluted to the concentrations used here, has no effect on the cardiac neuromuscular system (Stevens et al., 2009). This solution was stored as a stock solution at −20°C. Just before use, an aliquot was thawed and diluted to the working concentration in saline. After a temperature ramp in control saline was completed, peptide was perfused through the whole heart preparation for a 15 min acclimation period at baseline temperature before a temperature ramp was repeated in the presence of 10^–8^ M SGRN or 10^–7^ M myosuppressin. These experiments were conducted using the whole heart in summer lobsters that had been acclimated in our tanks for less than two weeks; the crash temperatures in control saline were noticeably higher than in other experiments.

### 2.10. Data analysis

Recordings of muscle contractions in whole heart, semi-intact heat, and stimulated preparations were analyzed for contraction amplitude and frequency. Recordings of ganglion bursting activity in semi-intact and isolated CG preparations were analyzed for burst frequency, burst duration, and duty cycle (defined as the burst duration over cycle period). Contraction parameters were determined using the built-in functions in Spike2. Bursting parameters were calculated using scripts for Spike2 written by Dr. Dirk Bucher (Rutgers University and New Jersey Institute of Technology).

To characterize the pattern of changes recorded as the temperature was increased toward crash temperature, we divided the entire temperature range into 1°C bins and averaged each parameter across all bursts within that temperature bin for each preparation. Values plotted at each temperature thus reflect the values for all cycles in a 1°C range around that value (e.g., the binned value at 12°C includes all cycles in which the temperature was 11.501°C through 12.500°C.) To enable us to pool data from multiple individual hearts or cardiac ganglia, each of which had different starting values for all parameters, we normalized all values for a given preparation to the value recorded at ∼8°C, before the start of the temperature ramp, in that preparation. Pooled data for bursting and contraction parameters are thus shown as normalized averages from multiple preparations. Crash temperatures were not normalized.

All data sets were tested for normality to determine the statistical tests to be used. For all student’s *t*-tests, we used a Shapiro–Wilk test to determine sample normality (particularly when sample sizes were small), and when appropriate, a Levene’s test to assess equal variance between groups. When normality could either not be assessed or when distributions failed a Shapiro–Wilk test, we used a Wilcoxon Sign-Rank test for paired data and a Friedman test with Dunn’s multiple comparisons for repeated measures data involving more than two sets of values. These tests were carried out in both MATLAB (Mathworks, Nantucket, MA, USA) and GraphPad Prism (Dotmatics, Boston, MA, USA).

When comparing the responses to LNA perfusion through the whole heart preparations (Figure 9A), we used a Wilcoxon Sign Rank test to determine if there was an increase in crash temperature for preparations whose contraction frequency was increased by LNA. We also used a Wilcoxon Sign Rank test to determine if there was a decrease in crash temperature for preparations whose contraction frequency was decreased by LNA. Because our sample size was small, we bootstrapped the data by pooling all values within each group (control vs. increased frequency and control vs. decreased frequency) and for each comparison, we randomly distributed the data (with replacement) into two groups and compared the newly generated groups with a Wilcoxon Sign Rank test. This process was repeated 10,000 times. All tests and bootstrapping were carried out in MATLAB (Mathworks, Nantucket, MA, USA).

When we compared the correlation between the change in crash temperature as a function of the change in heartbeat frequency between control and LNA conditions (Figure 10B), we used a Spearman rank correlation test. We used the same test to assess whether or not the contraction frequency in the control condition predicted the crash temperature of that preparation in LNA (Figure 10C), as well as to ask whether contraction frequency at baseline temperature predicted the crash temperature of that preparation in saline for a separate group of whole heart preparations (Figure 11). In both cases, analyses were carried out in MATLAB (Mathworks, Nantucket, MA, USA).

## 3. Results

### 3.1. The cardiac neuromuscular system maintains rhythmicity across a wide temperature range

We used two simultaneous approaches to examine how the cardiac neuromuscular system responds to changes in temperature in semi-intact preparations: (1) We recorded cardiac muscle contractions using a force transducer (Figure 1A, upper trace). In these recordings, each upward deflection corresponds to a single muscle contraction and each downward deflection corresponds to cardiac muscle relaxation. (2) We simultaneously monitored the bursts of action potentials produced by the CG neurons (Figure 1A, lower trace), using a suction electrode to record extracellularly from the CG nerve (Methods). These action potentials are generated by the five large cells (LCs; motor neurons). Rhythmicity in the LCs is coordinated largely by the four small cells (SCs; pacemaker neurons) and is maintained and stabilized by the recurrent synaptic connections between these two groups of neurons (Figure 1C), as well as by some intrinsic properties of the LCs. Because the heart is dissected from the animal in these experiments and because an incision is made in the heart muscle in order to record from the CG, these preparations are referred to as “semi-intact.”

**FIGURE 2:**
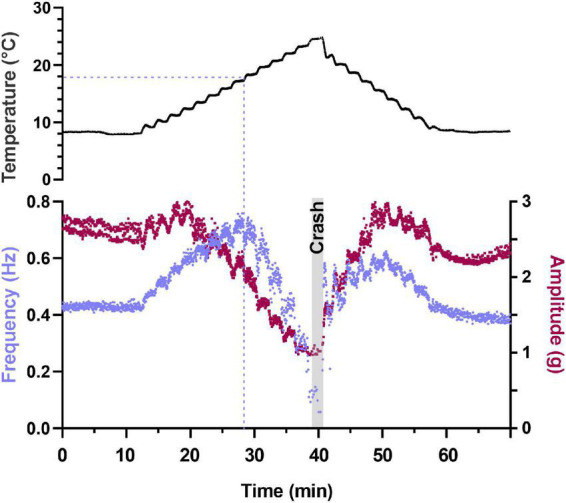
Depiction of changes in heartbeat parameters as temperature is increased to its crash temperature and then decreased to baseline. Plot of two parameters that characterize the heartbeat as temperature is increased to crash in a semi-intact preparation. Instantaneous contraction frequency is plotted on the left y-axis (purple) and contraction amplitude (force) on the right y-axis (maroon) as a function of time to crash. Each data point represents a single heartbeat. Temperature steps are indicated above the plot (1.25°C increments) and the gray bar labeled “Crash” denotes the time and temperature at which the preparation crashed (fewer than two beats in 30 s). Note that contraction amplitude decreased as temperature was increased above ∼12°C and frequency initially increased with increased temperature. This is followed by a decrease in both parameters (amplitude decreases at temperatures greater than ∼14°C and frequency decreases at temperatures greater than ∼20°C). Also note that when temperature is stepped back to 8°C, we observe a mirrored response in both frequency and amplitude relative to the temperature ramp to crash.

Semi-intact preparations maintained rhythmic cardiac contractions as temperature was increased, until a critical temperature was reached. At this critical temperature, the muscles stopped contracting and the cardiac ganglion failed to generate rhythmic bursts of action potentials. So long as activity was recovered when temperature was decreased back into the permissible range, this loss of functional activity was called a “crash” (Tang et al., 2012; Rinberg et al., 2013). Figure 2 shows an example of this crash and recovery in a single preparation. Here we have plotted the instantaneous frequency and amplitude of each heart contraction as a function of time while temperature was being increased from ∼8°C to ∼25.5°C (the crash temperature for this preparation). While our results focus primarily on the effects of increasing temperature on this circuit-muscle effector system, it is important to note that the neuromuscular system did recover when temperature was decreased from the crash temperature, as is shown in Figure 2. In this example, the frequency and amplitude of the heart contractions initially increased as temperature was increased. This increase was followed by a decrease in both parameters as the temperature was increased to the crash point (∼25.5°C). Notably, the temperature dependencies of frequency and amplitude were not identical. In this preparation, for example, the decrease in amplitude started at ∼13°C, whereas frequency did not start to decrease until the temperature had reached ∼17°C. Decreasing temperature back to ∼8°C caused an approximately mirrored response for both frequency and amplitude. Although some differences can be observed, this may be due to a slower time course in increasing saline temperature compared to the faster decrease back to baseline temperature.

**FIGURE 3:**
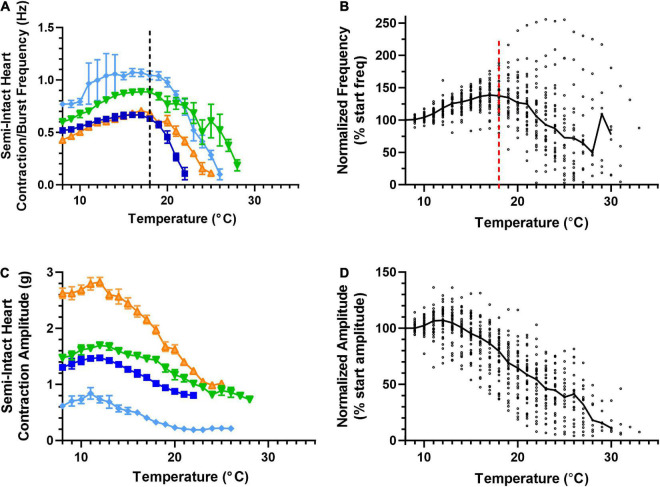
Cardiac neuromuscular system responses to an increasing temperature ramp: contraction frequency and amplitude. Values show data averaged in 1°C bins, with the data plotted at the center of each bin. **(A)** A plot of heart contraction frequency in response to a temperature ramp from 8°C to crash temperature for four semi-intact preparations (each shown in a different color). Note that at ∼18°C (dashed line), contraction frequency began to decrease in each of these example preparations. **(B)** A plot of heart contraction frequency normalized to the contraction frequency at 8°C in response to a temperature ramp from 8°C to crash temperature for all semi-intact preparations. (*N* = 35 until the first preparation crashes at 19°C; *N* decreases from that temperature on). **(C)** Plot showing contraction amplitude (force, grams) as a function of temperature for the same four semi-intact preparations plotted in panel **(A)**. Note that contraction amplitude decreased as temperature was increased above ∼12°C. **(D)** A plot of contraction amplitude as a function of temperature, with the contraction amplitude for each preparation at each temperature step normalized to the contraction amplitude at 8°C (*N* = 35 until the first preparation crashes at 19°C; *N* decreases from that temperature on). All data points in panels **(A,C)** show the mean ± SD for a given parameter at each temperature step. In panels **(B,D)**, the solid back line connects median normalized contraction frequency and amplitude, respectively, at each temperature step. Medians are omitted when *N* ≤ 5.

**FIGURE 4:**
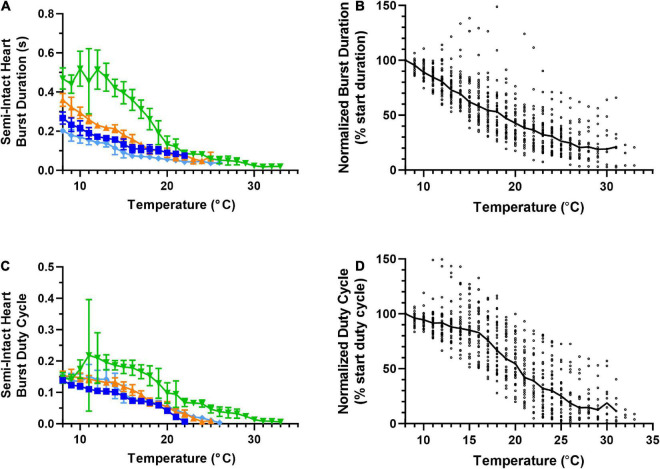
Responses of semi-intact cardiac neuromuscular system to an increasing temperature ramp: cardiac ganglion burst duration and duty cycle. Values show data averaged in 1°C bins, with the data plotted at the center of each bin. **(A)** A plot of cardiac ganglion burst duration within semi-intact hearts as a function of temperature for the same four semi-intact preparations in Figures 3A, C. CG burst duration decreases rapidly with temperature, and eventually stabilizes at a floor duration. **(B)** A plot of CG burst duration normalized to the duration at 8°C for all semi-intact preparations, showing the decrease in contraction duration across all temperatures (*N* = 35 until the first preparation crashes at 19°C; *N* decreases from that temperature on). **(C)** Plot of CG burst duty cycle as a function of temperature for the same four preparations plotted in panel **(A)**. CG burst duty cycle decreases across the entire range of temperatures tested. **(D)** A plot of CG burst duty cycle as a function of temperature, with the duty cycle for each preparation at each temperature step normalized to the burst duty cycle at 8°C (*N* = 35 until the first preparation crashes at 19°C; *N* decreases from that temperature on). Duty cycle decreases with increasing temperature over the entire range of temperatures. All data points in panels **(A,C)** show mean ± SD for a given parameter at each temperature step. In panels **(B,D)**, the solid back line connects median normalized burst duration and duty cycle, respectively at each temperature step. Medians are omitted when *N* ≤ 5.

We measured four parameters as we increased temperature to the crash point in a set of semi-intact preparations (Methods). We measured the frequency of the heartbeat/CG bursts and heartbeat force of 35 hearts (Figure 3) and the motor neuron burst duration and duty cycle of 35 hearts (Figure 4). Thirty of the hearts recorded are in both data sets, but due to the varying qualities of recordings, the last 5 hearts in each group are from separate sets of five hearts each. Because there is a one-to-one relationship between a burst of action potentials from the CG and the subsequent heart contraction, burst frequency and contraction frequency are synonymous (Figure 1A; see dashed line). They are thus depicted in a single plot. Although the baseline frequency and the details of the pattern of changes varied across individual preparations, as can be seen in the four example preparations shown in Figure 3A, the frequency initially increased with increasing temperature until approximately 18–19°C (mean peak frequency was recorded at 18.03 ± 2.88°C; *N* = 35), and then began to decrease until a critical temperature was reached and the preparations crashed. A similar pattern was observed across the population of hearts studied here. Figure 3B shows that as temperature was increased until each preparation crashed, median contraction frequency increased until ∼18°C, at which point median contraction frequency began to decrease. When temperature was increased beyond ∼26°C, the fraction of crashed preparations substantially increased (at 26°C, *N* = 13/35, at 28°C *N* = 26/35). Medians are not shown for temperatures at which five preparations or fewer remained in the testing set, i.e., for temperatures at or higher than that at which all but five of the preparations had crashed.

The force (measured here as contraction amplitude) generated by the heartbeat was measured at each temperature (Figure 3C); the example preparations plotted here show that contraction amplitude varied greatly across preparations. As was the case with frequency, heartbeat force began to decrease when temperature was increased beyond a certain temperature. However, this decline in force generally began at a lower temperature (declining above ∼12°C in most preparations). Previous studies show that a functional relationship exists between CG burst frequency and heartbeat amplitude (Mahadevan et al., 2004; Worden et al., 2006; Wiwatpanit et al., 2012; Williams et al., 2013). Although both frequency and amplitude eventually decreased as temperature was increased, the decrease in contraction frequency occurred at temperatures higher than the decrease in contraction force, as noted above (Figures 3A, B). This was true regardless of initial contraction strength, suggesting that either changes other than contraction frequency [e.g., duty cycle, which is an important component of the neuromuscular transform in the lobster cardiac neuromuscular system (Williams et al., 2013)] or changes at the periphery also play a role in the response to changing temperature.

Unlike heartbeat force and frequency (Figure 3), the duration of CG bursts (Figures 4A, B) decreased with each increased temperature step until around 24°C, at which point burst duration appears to asymptote, with a minimum burst duration being maintained until the preparations crashed. Interestingly, the decrease in duty cycle between 8°C and 16°C was gradual (∼10%; Figure 4D) relative to the increase in contraction frequency (∼40%; Figure 3A), indicating that the initial increase in contraction frequency was due to a simultaneous decrease in burst duration (Figure 4A) and interburst interval (not shown). As temperature was increased beyond ∼16°C, we observed a clear decrease in duty cycle (Figures 4C, D) along with burst duration (Figures 4A, B). However, between 13°C and 18°C, contraction frequency increased with increased temperature (Figures 3A, B), meaning that the observed decrease in burst duration (Figure 4A) was occurring at a slower rate than the decrease in duty cycle (Figure 4C). Between 25°C and 30°C, the decrease in burst duration began to asymptote (Figure 4B) along with duty cycle (Figure 4D). This explains why contraction frequency decreased in this warmer temperature range (Figures 3A, B). This is certainly the case at temperatures greater than ∼25°C, where burst duration is brief but stable (Figures 4A, B), and duty cycle and frequency continue to decrease until the crash temperature is reached (Figures 4C, D and Figures 3A, B, respectively). At temperatures greater than 30°C, too few preparations were still active to decipher any kind of trend.

### 3.2. The isolated CG crashes at a higher temperature than the semi-intact neuromuscular system

**FIGURE 5:**
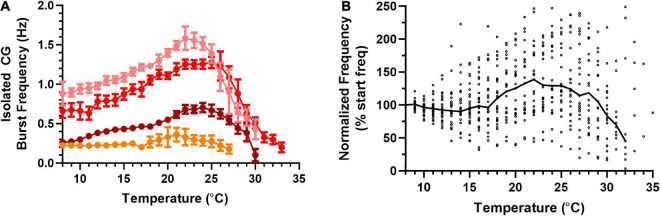
Frequency of bursts in an isolated cardiac ganglion increases, then decreases in response to an increasing temperature ramp. Values show data averaged in 1°C bins, with the data plotted at the center of each bin. **(A)** Plot of CG burst frequency as a function of temperature for four example preparations as temperature was increased from 8°C to crash temperature. Note that there no longer exists an inflection point in burst frequency at 18°C as was the case for contraction frequency. Although three of the preparations show clear increases followed by decreases in frequency, the change from increase to decrease occurred at different temperatures in each preparation. All data points show the mean ± SD for burst frequency at each temperature step. **(B)** Plot of normalized burst frequency as a function of temperature, with the burst frequency of each preparation at each temperature step normalized to the burst frequency at 8°C (*N* = 25 until the first preparation crashes at 20°C; *N* decreases from that temperature on, until *N* = 1 at 35°C). The solid back line connects median normalized burst frequency at each temperature step. Medians are omitted when *N* ≤ 5.

To better understand the biological processes that enable the cardiac neuromuscular system to maintain output across this wide range of temperatures, we asked how the temperature resilience of the isolated nervous system, which lacks any feedback input, compared to that of the semi-intact heart. Similar to the semi-intact experiments, the temperature of isolated CGs was stepped from ∼8°C to each preparation’s critical temperature (Figure 5A). Across isolated CGs, we observed an increase in variability in burst frequency as temperature was increased. However, median burst frequency across preparations did not increase until temperature was increased above ∼17°C (Figure 5B). Interestingly, the isolated CGs maintained this trend of increasing cycle frequency with increased temperature until ∼22°C, before the cycle frequency began to decrease with increased temperature (*N* = 25). These isolated CGs were extracted from a subset of the same hearts as the semi-intact preparations (Figures 3, 4). Thus, it appears that the temperature dependencies of the processes governing cycle frequency in the intact neuromuscular system differ from those in the isolated CG (Figures 3A, B vs. Figures 5A, B). Although we measured the burst duration and duty cycle at each temperature step for each of the isolated CG preparations, when comparing these parameters, the isolated CGs did not substantially differ from the semi-intact preparations (data not shown).

**FIGURE 6:**
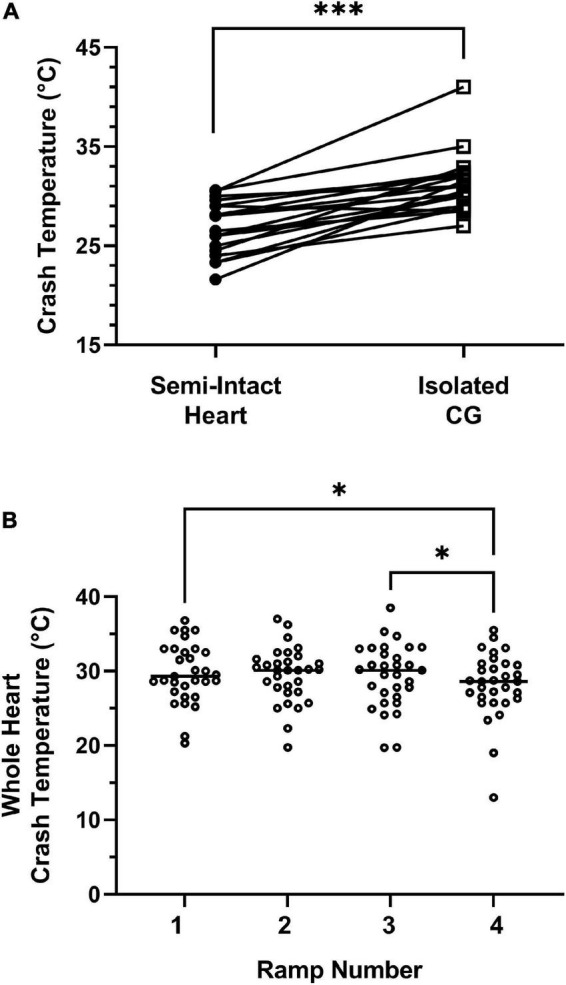
Isolated ganglia crash at a higher temperature than semi-intact preparations. **(A)** The crash temperatures of the isolated ganglia (ICG; 31.0°C; median) were higher than those of the semi-intact preparations (26.5°C; median); Wilcoxon Sign Rank test: *p* < 0.001; Shapiro–Wilks test for normality: Semi-intact heart, *p* = 0.45; ICG, *p* = 0.005; *N* = 19. **(B)** Crash temperature does not increase with repeated ramps, as seen in this comparison of four repeated temperature ramps in whole heart preparations. Although there were significant differences in crash temperature across hearts (Friedman repeated measures test: *p* = 0.008; *N* = 31), this reflected differences only between Ramps 1 and 4 (Dunn’s multiple comparison test, *p* = 0.047; *N* = 31) and Ramps 3 and 4 (Dunn’s multiple comparison test, *p* = 0.03; *N* = 31); Shapiro–Wilks test for normality: Ramp 1, *p* = 0.44; Ramp 2, *p* = 0.52; Ramp 3, *p* = 0.97; Ramp 4, *p* = 0.01; *N* = 31. In both of these cases, the median recorded for the later ramp, i.e., Ramp 4, was lower rather than higher than the median crash temperature of the earlier ramp. (Median for Ramp 1: 29.3°C; median for Ramp 3: 30.1°C; median for Ramp 4: 28.6°C). Bars in panel **(B)** indicate the median crash temperature. *Indicates *p* < 0.05; ^***^Indicates *p* < 0.001.

Because the overall patterns of changes in CG activity were similar, but the temperatures at which the pattern changed appeared to differ between isolated CGs and non-isolated CGs within semi-intact preparations, we compared the crash temperatures of the non-isolated CGs to those of the isolated ganglia (Figure 6). After assessing the crash temperature in the semi-intact preparation, the CG was subsequently dissected out of the heart (Methods). In all but one preparation, the isolated CG crashed at a higher temperature than semi-intact heart from which it had been removed (Figure 6A, semi-intact vs. isolated CG; Wilcoxon Sign Rank test: *p* < 0.001, *N* = 19).

We initially postulated that the difference in crash temperatures in the two preparations might be due to some type of temperature adaptation, as we could not counter-balance the experimental order of each temperature ramp (i.e., we cannot re-insert a dissected nervous system back into the heart). To address this issue, we subjected 31 whole heart preparations to four sequential temperature ramps (Figure 6B). Although there were some differences in the crash temperatures with repeated ramps (Friedman test, *p* = 0.008, *N* = 31), these differences involved only the fourth ramp (Friedman/Dunn’s multiple comparisons test: Ramp 1 vs. 4: *p* = 0.047; Ramp 3 vs. 4: *p* = 0.030, *N* = 31). That is, there were no changes in crash temperature between ramps 1, 2, and 3. Moreover, the crash temperature for Ramp 4 was lower than that of previous ramps, rather than higher, as would be expected if the repeated heating were responsible for the difference in the crash temperatures of the ICG relative to the semi-intact heart.

There are a number of explanations that may address why the semi-intact cardiac neuromuscular system crashed at lower temperatures than the isolated CG: (1) At high temperatures (>30°C) the cardiac muscles may fail to contract even though the CG neurons are rhythmically producing action potential bursts. (2) The absence of NO feedback in the isolated CG may enable the ganglion to reach higher temperatures before crashing. For example, the nitric oxide (NO) production in the cardiac muscles that inhibits CG output (negative feedback pathway) may increase with increased temperature and inhibit CG action potentials prior to neuron crash point (i.e., the crash point observed in the isolated CG). (3) A non-intuitive change in the stretch feedback pathway may take place as a consequence of increased temperature. This last possibility is non-intuitive because stretch feedback most often produces excitatory drive (positive feedback) to the CG, such that muscle stretch promotes motor neuron activity. Therefore, the next several experiments detailed here address the hypotheses concerning the temperature tolerance of the cardiac muscles and NO feedback onto the CG neurons.

### 3.3. Semi-intact crashes are not due to muscle contraction failure

We first assessed whether the relatively cooler crash temperature observed in the semi-intact neuromuscular systems (Figure 6A) was in fact due to a failure of the cardiac muscle to contract at high temperatures (>20°C). For these experiments, we dissected out most of the CG from the heart (all neurons; Methods) while leaving a length of the anterior lateral nerve intact such that we could extracellularly stimulate the motor nerve *via* a suction electrode (Figure 1B; Methods). With this setup, we recorded the force generated by each cardiac muscle contraction in response to stimulation, which generated constant bursts of action potentials (frequency, duration, duty cycle). We stimulated the motor nerve at progressively increased temperatures in 1.5°C increments between baseline temperature (∼8–9°C) and the crash temperature of that heart when previously tested in the semi-intact configuration.

Although endogenously the CG spontaneously produces bursts of action potentials continuously, we delivered our stimuli in bouts of 15 bursts, followed by a pause of 1 min; continual stimulation results in nerve failure within a relatively short time (Stevens et al., 2009). Because of this, we can see the patterns of facilitation and defacilitation that occurred with repeated stimuli. Although the dynamics of successive contractions differed across temperature, the muscles produced contractions in response to stimulation across the entire temperature range in all preparations.

For these experiments, we first assessed the crash temperature in each preparation before extracting the nervous system from the heart (Methods). After removing the CG from a given heart, we measured muscular responses to nerve stimulation at baseline and in response to a temperature ramp that rose to the previous crash temperature for that preparation. All heart muscles were able to contract in response to stimulation at the same temperature that the intact preparation crashed (*N* = 9). Therefore, crashes are not due to a failure of muscle contraction.

**FIGURE 7:**
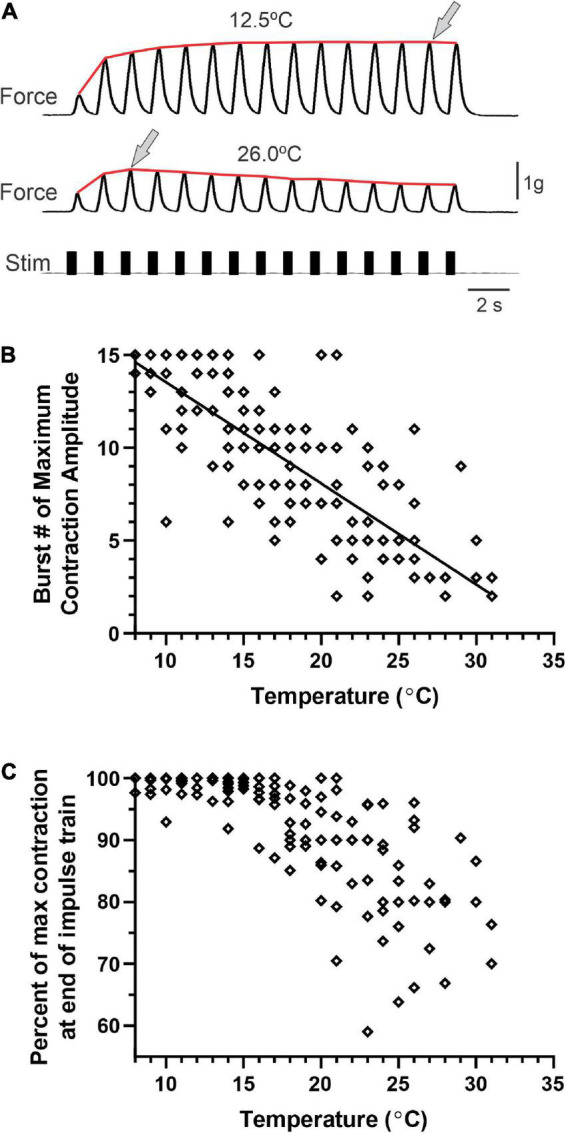
Cardiac muscle contractions persist at high temperatures, although facilitation is altered by temperature. **(A)** Example recordings of evoked muscle contractions at 12.5°C (top trace) and 26°C (middle trace) in response to exogenous nerve stimulation (lower trace). Arrows indicate the contraction of maximum amplitude in each recording. The red line shows the envelope of the contractions at each temperature. Both recordings are from the same preparation. **(B)** Plot of the burst number at which evoked contractions reach maximum amplitude for a train of stimulated contractions at each temperature (1.5°C increments) between 8°C and 31°C. Note that the contraction at which maximum amplitude is reached within the train of stimuli decreases with increased temperature (*R*^2^ = 0.63, *p* < 0.001). **(C)** Contractions showed more defacilitation over the train of contractions at higher temperatures. Plot of the percentage of the maximum contraction amplitude that remained at the end of the train of stimuli [i.e., 100*(Amplitude last contraction/amplitude max contraction)]; *N* = 9.

Figure 7A shows two example muscle tension recordings from the same preparation, one at 12.5°C and one at 26°C, to the same stimulus. For the responses recorded at 12.5°C (Figure 7A, top trace), contraction force increased noticeably over the first 4–5 stimuli. The remaining 10–11 stimuli in the train resulted in contractions that were more forceful but stabilized by the end of the train of stimuli. In contrast, at 26°C (Figure 7A, bottom trace), the largest muscle contraction was in response to the third stimulus; contraction force then decreased over the remainder of the train of stimuli.

Because this is the first study to examine the temperature dependence of facilitation and defacilitation in the cardiac nervous system of *Homarus*, and because these relationships are clearly different at low and high temperatures, we analyzed two aspects of the muscle facilitation/defacilitation relationship. First, to examine the temporal relationship of the facilitation vs. defacilitation of muscle contraction, we plotted the number of the stimulus (indexed to 1) that generated the maximum contraction amplitude for each temperature.

Each stimulus train generated fifteen stimuli (Figure 7A, stim. trace), so values on this plot are bounded between 1 and 15. As temperature was increased, the maximum contraction amplitude was reached earlier in the train of stimuli (Figure 7B); facilitation occurred over fewer stimuli, with contraction amplitude decreasing later in the train of repeated stimuli, as seen in the example in Figure 7A. Note that although the initial response amplitude in the example traces in Figure 7A was relatively small, this was not always the case. Second, to examine the extent to which facilitation vs. defacilitation dominated the stimulus bout, we compared the ratio of response magnitude (force) between the last stimulus response in each stimulation train and the maximal response (Figure 7C). For example, the responses recorded at 12.5°C show that the smallest force generated occurred early in the stimulus train (i.e., 1st stimulus here), and the next to final stimulus generated the maximum force. In contrast, at 26°C, the third stimulus response was the largest, while the final response was one of the smallest. This is reflected in the plot shown in Figure 7C, where the ratio of maximum contraction to final contraction decreased as temperature was increased. At the higher temperatures, defacilitation dominated, especially later in the train of stimuli, whereas there was little or no defacilitation at the lower temperatures.

### 3.4. Nitric oxide decreases crash temperature in the isolated CG

**FIGURE 8:**
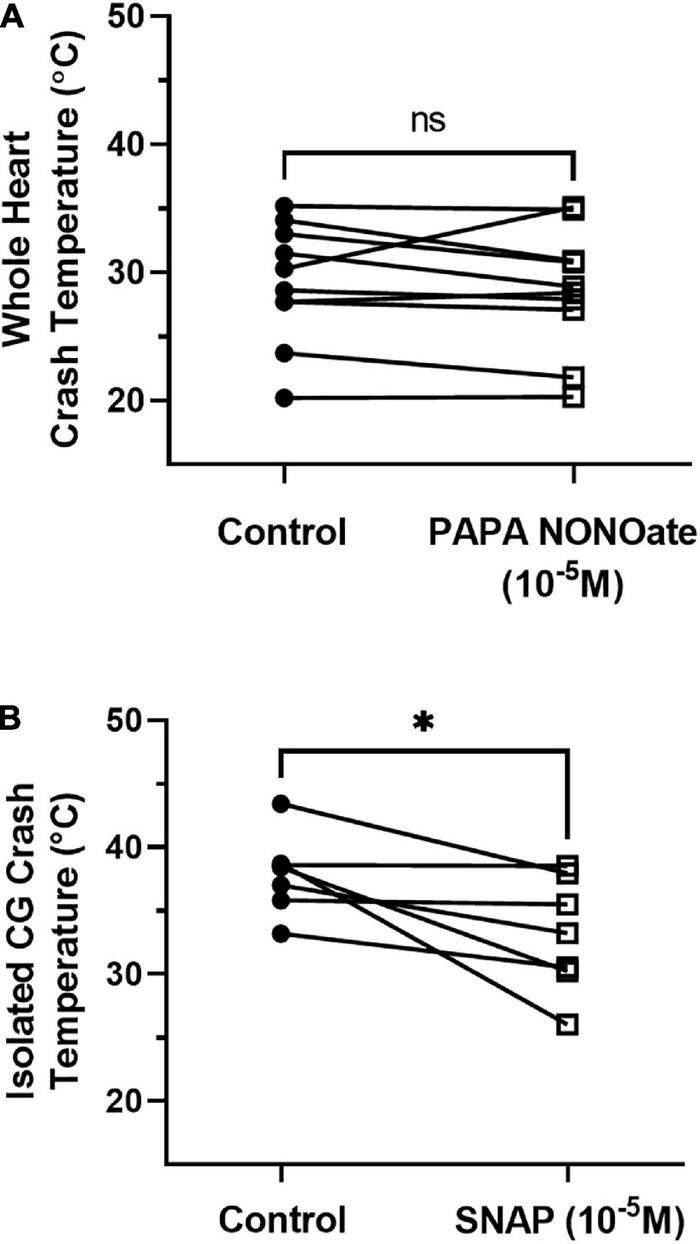
NO donors differently affect the crash temperature of whole heart and ICG preparations. **(A)** Plot of crash temperature for whole hearts perfused with normal saline compared to the same hearts perfused with saline and the NO donor PAPA NONOate (10^– 5^ M). PAPA NONOate did not alter crash temperature; paired *t*-test: *p* = 0.4, *N* = 10; Shapiro–Wilks test: saline: *p* = 0.69, *N* = 10; NONOate: *p* = 0.47, *N* = 10. **(B)** Comparison of crash temperature between isolated cardiac ganglia superfused with normal saline and ganglia superfused with saline and the NO donor SNAP (10^– 5^ M) within the same set of preparations. Preparations exposed to SNAP crashed at a lower temperature than controls; paired *t*-test, *p* = 0.032, *N* = 7; Shapiro–Wilks test: saline: *p* = 0.49, *N* = 7; SNAP: *p* = 0.53, *N* = 7. *Indicates *p* < 0.05, ns indicates not significant.

We next assessed how the presence of NO affected the crash temperature of both the intact cardiac neuromuscular system (whole heart preparation) and the isolated CG. NO is synthesized in the cardiac muscle and then diffuses across neuron membranes to affect activity (Mahadevan et al., 2004). The addition of NO (*via* an NO donor) to the isolated CG usually decreases burst frequency by increasing the interburst interval (Mahadevan et al., 2004; Goy, 2005). Consistent with these observations, when we perfused an NO donor (PAPA NONOate) through the whole heart, we recorded a small (∼10%) decrease in frequency (control saline: mean 0.502 ± 0.112 SD; PAPA NONOate: mean 0.455 ± 0.089 SD; paired *t*-test, *p* = 0.04, *N* = 10). However, we observed no change in crash temperature in the presence of PAPA NONOate (Figure 8A; paired *t*-test, *p* = 0.4, *N* = 10). In contrast, when we superfused an NO donor (SNAP) over the isolated ganglion, which, without the associated cardiac muscles, has no other source of NO, we observed a significant decrease in crash temperature (Figure 8B; paired *t*-test, *p* = 0.015, *N* = 8). NO application was sufficient to decrease the crash temperature in the isolated CG (Figure 8B), and it also decreased the CG crash temperatures into the same range observed for whole heart preparations (i.e., compare Figure 8B “SNAP” to Figure 8A “Control”; Independent Sample *t*-test: *p* = 0.065, *N* = 8, 10; Shapiro–Wilks test: *p* = 0.69, *N* = 8; *p* = 0.63, *N* = 10; Levene’s test: *p* = 0.9, *N* = 8, 10). However, the magnitude of the difference between the crash temperature in the whole heart preparation and isolated CG (Figure 6A) and the magnitude of the difference between the crash temperature in control (saline) and SNAP (Figure 8B) was not significantly different (Independent Sample *t*-test; *p* = 0.5, *N* = 8, 19; Shapiro–Wilks test: *p* = 0.29, *N* = 8; *p* = 0.38, *N* = 19; Levene’s test: *p* = 0.035, *N* = 8, 19).

**FIGURE 9:**
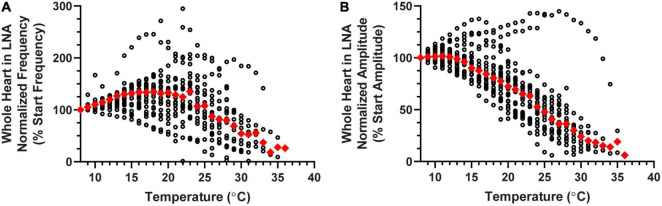
Whole heart responses to a temperature ramp in LNA. Plot of **(A)** normalized contraction frequency and **(B)** normalized contraction amplitude measured in the whole heart as a function of temperature during LNA perfusion. Contraction frequency **(A)** increases until about 20°C, at which point it begins to decrease with increased temperature. Contraction amplitude **(B)** began to decrease at temperatures greater than 13°C. Red triangles represent the median response at each temperature step for panels **(A,B)**.

**FIGURE 10:**
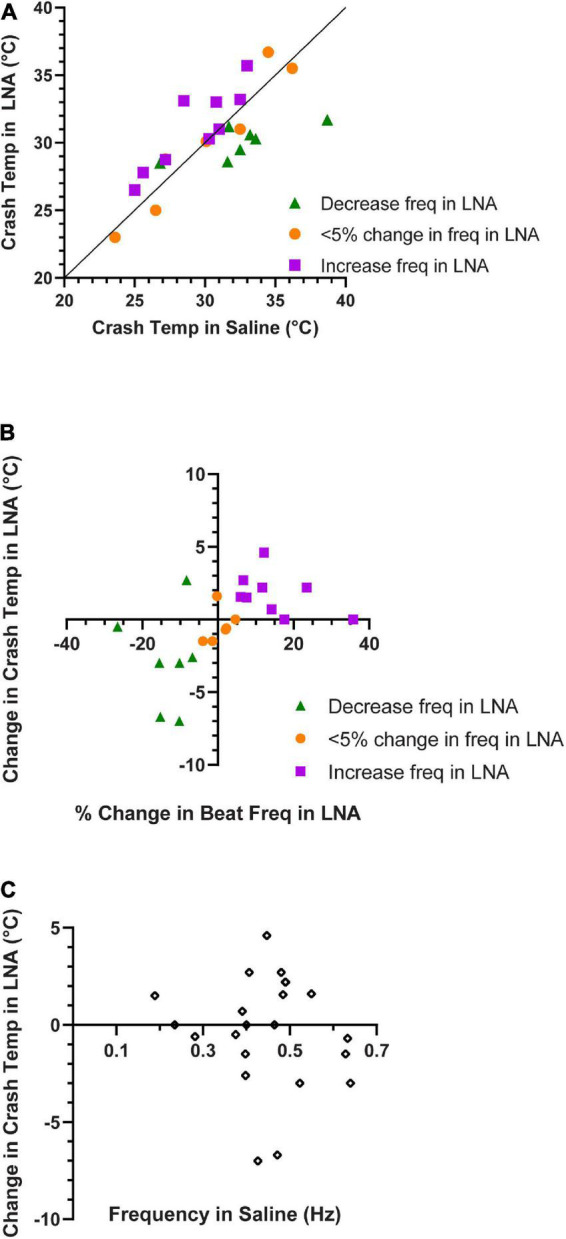
Contraction frequency in LNA correlates with crash temperature. **(A)** Comparison of crash temperatures between whole heart preparations perfused with saline vs. L-nitroarginine (LNA) for the same preparations. The unity line indicates the value at which crash temperature is identical in saline and LNA. Preparations in which LNA led to an increase in contraction frequency are shown as green triangles (Wilcoxon Sign-Rank test; *p* = 0.047, *N* = 7); those in which LNA did not change the contraction frequency are depicted as orange circles (Wilcoxon Sign-Rank test; *p* = 0.56, *N* = 6), and those in which LNA led to and a decrease in contraction frequency are shown as purple squares (Wilcoxon Sign-Rank test; *p* = 0.016, *N* = 9). Note that most of the preparations in which frequency decreased in LNA are above the line (increased crash temperature in LNA), while most of those in which frequency increased in LNA are below the line (decreased crash temperature in LNA). **(B)** Plot of whole heart crash temperature in LNA as a function of their percent change in contraction frequency. Spearman’s Rank Correlation test: ρ = 0.64, *d* = 3.74, *p* = 0.0013, *N* = 22. **(C)** The change in crash temperature (LNA crash temperature–saline crash temperature) did not change as a function of the initial contraction frequency, Spearman’s Rank Correlation test: ρ = –0.25, *d* = –1.14, *p* = 0.26, *N* = 22.

**FIGURE 11:**
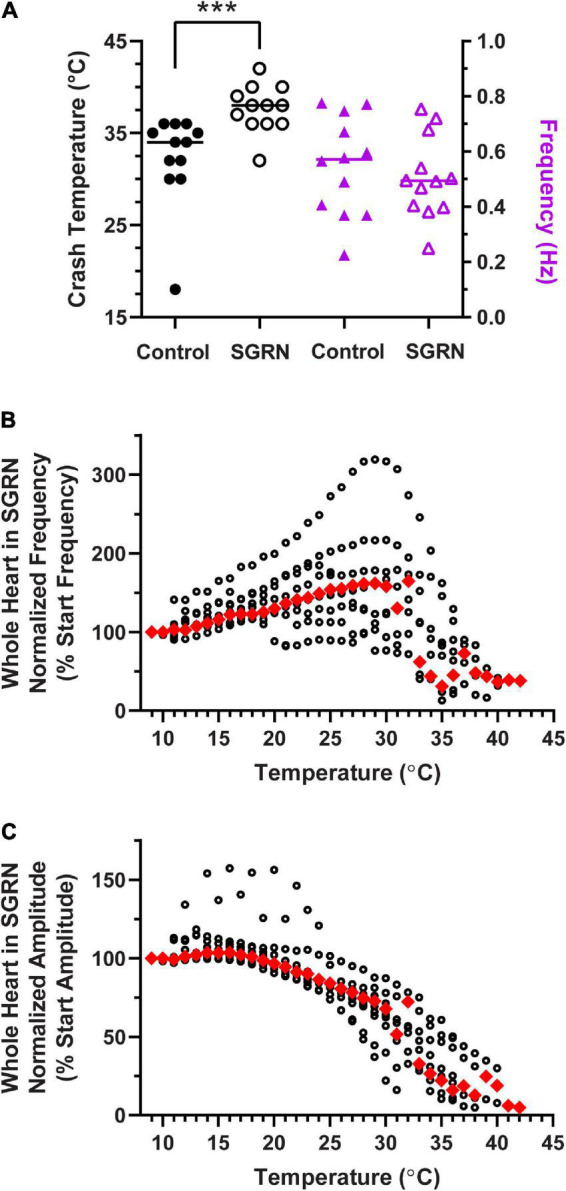
Modulation of the cardiac neuromuscular system by SGRN increases crash temperature. **(A)** Comparison of crash temperatures between whole heart preparations perfused with saline (34°C, median) vs. 10^– 8^ M SGRN (38°C, median) in the same hearts. SGRN extended the temperature tolerance of these hearts (Wilcoxon test; *p* = 0.0005, *N* = 12). However, although SGRN generally excites cardiac activity, at this concentration, contraction frequency increased in only about half of the hearts, while it decreased in the other half, so that the average change in contraction frequency was not altered by SGRN [right y-axis; saline: 0.57 Hz, SGRN: 0.49 Hz (median frequencies); Wilcoxon test; *p* = 0.47, *N* = 12]. **(B)** Normalized whole heart contraction frequency as a function of temperature during SGRN perfusion. Note that contraction frequency increased until about 29°C, which is when preparations began to crash. **(C)** Normalized whole heart contraction amplitude as a function of temperature during SGRN perfusion. Contraction amplitude with increased temperature until ∼19°C, at which point, it decreased until crash. Red triangles represent the median response at each temperature step for panels **(B,C)**. ***Indicates *p* < 0.001.

Although different NO donors were used in the whole heart and in the isolated CG, we do not believe that using different NO donors affected our results. For each molecule of PAPA NONOate, two molecules of NO are released (Hrabie et al., 1993; Keefer et al., 1996). Because SNAP only dissociates NO with a 1:1 ratio (Singh et al., 1996), our effective concentrations of NO were 2 × 10^–5^ M using PAPA NONOate and 1 × 10^–5^ M using SNAP. Because the PAPA NONOate application did not have an effect on the whole heart crash temperature, it is unlikely that SNAP would affect the crash temperature.

### 3.5. Blocking nitric oxide in the whole heart decreases crash temperature

Although the addition of NO to the isolated ganglion is sufficient to decrease the crash temperature of the isolated CG, we also wanted to test whether blocking NO synthesis in the cardiac muscles would increase the crash temperature of the whole heart preparation. To do this we perfused whole heart preparations with L-Nitroarginine (LNA) to block NO synthase activity.

As previously mentioned, NO typically decreases CG burst frequency and therefore heartbeat frequency. Blocking NOS should therefore result in an increase in heart rate. However, when LNA was perfused through the whole heart in a set of hearts that were not subjected to temperature ramps, we only observed such an increase in heart rate ∼50% of the time (increased heart rate: One-Sample Wilcoxon Sign-Rank test: *p* < 0.001, *N* = 18; decreased heart rate: One-Sample Wilcoxon Sign-Rank test: *p* < 0.001, *N* = 12; five preparations had a less than 5% deviation from control–too few to do statistics; data not shown). In a separate set of experiments, LNA was perfused through the whole heart while temperature was increased to crash point. Although LNA did change the baseline frequency in many of these preparations (see below), the patterns of change in both normalized contraction frequency (Figure 9A) and normalized contraction amplitude (Figure 9B) during temperature ramps were similar to the patterns recorded in response to temperature ramps during normal saline perfusion (Figures 3B, D). Specifically, contraction frequency initially increased as temperature was increased, then decreased until it reached crash point. Contraction amplitude was stable during the initial few temperature steps, then gradually decreased over the course of the temperature increase. As can be seen in Figure 9, there was some variability among preparations, but the median patterns were similar to those recorded in normal saline.

Similar to our previous findings, ∼30% of the hearts perfused with LNA showed an increase in heart rate, while the heart rate decreased or did not change in the remaining preparations. Interestingly, when LNA resulted in an increase in beat frequency, these hearts crashed at a higher temperature than they did during saline perfusion (Figures 10A, purple squares; Wilcoxon Sign-Rank test; *p* = 0.047, *N* = 7). Consistent with these results, we found that the opposite was true for the ∼40% of hearts from this experiment whose response to LNA was a decreased heart rate. These hearts crashed at a lower temperature compared to saline perfusion (Figure 10A, green triangles; Wilcoxon Sign-Rank test; *p* = 0.016, *N* = 9). Because these sample sizes are small, we bootstrapped each LNA response data set (increased frequency: *p* = 0.018; decreased frequency *p* = 0.045; Methods). The remaining 30% of hearts showed < 5% change in contraction frequency in response to LNA, and the crash temperature in LNA was not different from saline (Figure 10A, orange circles; Wilcoxon Sign-Rank test; *p* = 0.56, *N* = 6). Figure 10B plots the change in crash temperature as a function of change in heartbeat frequency in the presence of LNA compared to control saline (Spearman’s Rank Correlation test: ρ = 0.64, *d* = 3.74, *p* = 0.0013, *N* = 22). Thus, LNA was able to extend the temperature tolerance in hearts that increase in heartbeat frequency when NO synthase is blocked, but it limited the temperature tolerance in preparations in which blocking NO synthase decreased the heart rate.

Figure 10A indirectly suggests that the crash temperature in control (saline) may predict the cardiac response to LNA. The control (saline) crash temperatures of most hearts in which beat frequency decreased in LNA were equivalent to the control crash temperatures of hearts in which LNA elicited an increase in beat frequency (Figure 10A, green triangles vs. purple squares) (independent sample *t*-test, *p* = 0.061, *N* = 7, 9; Shapiro–Wilks test: decreased freq. in LNA: *p* = 0.2, *N* = 7, increased freq. in LNA: *p* = 0.5, *N* = 9; Levene’s test: *p* = 0.82, *N* = 7, 9). There was no correlation between saline (control) crash temperature and LNA crash temperature (Spearman’s Rank Correlation test: ρ = 0.53, *d* = 2.07, *p* = 0.062, *N* = 13). Furthermore, there is no correlation between the initial heartbeat frequency at ∼8°C and the change in crash temperature in response to LNA (Figure 10C; Spearman’s Rank Correlation test: ρ = −0.25, *d* = −1.14, *p* = 0.26, *N* = 22).

It is possible that the change in contraction frequency in response to LNA was due to the baseline contraction frequency (e.g., LNA decreased contraction frequency in preparations with a high initial frequency). However, there was no difference between the baseline contraction frequency in preparations in which LNA increased contraction frequency compared to preparations in which LNA decreased contraction frequency (Wilcoxon Mann–Whitney test: *p* = 0.41, *N* = 7, 9; data not shown).

### 3.6. Neuromodulation can bi-directionally affect functional temperature range of the nervous system

Our findings from Figure 10 indicate that there may exist a bi-directional effect on crash temperature such that modulation that increases heartbeat frequency also increases the crash temperature and modulation that decreases heartbeat frequency, decreases the crash temperature. To test this hypothesis, we applied two different feedforward neuromodulators to the whole heart preparation, one that is known to increase heartbeat frequency and one that decreases it.

To assess whether neuromodulators that increase heartbeat frequency could extend the temperature tolerance of the cardiac neuromuscular system, we perfused the FLRFamide-like neuropeptide SGRNFLRFamide (SGRN) through the whole heart while temperature was increased to crash. SGRN does in fact increase the temperature tolerance of the whole heart (Figure 11A; Wilcoxon Sign-Rank test; *p* = 0.0005, *N* = 12). However, the responses of lobster hearts to 10^–8^ M SGRN were highly variable (Figure 11A, purple triangles); in some hearts, 10^–8^ M SGRN increased contraction frequency, while in others, it decreased contraction frequency, as has been seen previously (Dickinson et al., 2015). Because of this, the mean frequency at baseline temperatures (8–10°C) did not change in these experiments (Figure 11A, purple triangles; Wilcoxon Sign-Rank test: *p* = 0.47, *N* = 12). This suggests that increased temperature resilience is not simply a function of change in contraction frequency but is instead a response to modulation that presumably increases neuron excitability. In this case, SGRN activates the modulator-activated, voltage-sensitive current I_*MI*_, which is an inward current whose conductance increases across the voltage range in which cardiac neurons oscillate (Swensen and Marder, 2000; Dickinson et al., 2015). The increased excitability in response to SGRN is reflected here in the changes observed in normalized contraction frequency (Figure 11B) and normalized contraction amplitude (Figure 11C) as a function of temperature. Whole hearts perfused with SGRN increased in contraction frequency as temperature was increased until ∼29°C, compared to an inflection point at about 18°C in saline (Figure 3B). Moreover, even though the median contraction frequency began to decrease at ∼30°C, the median contraction frequency did not drop below 100% until preparations were nearing their crash temperatures (≥32°C). SGRN also extended the temperature range over which the normalized whole heart contraction amplitude remained ≥100% (∼19°C, Figure 11B vs. ∼14°C, Figure 3D).

**FIGURE 12:**
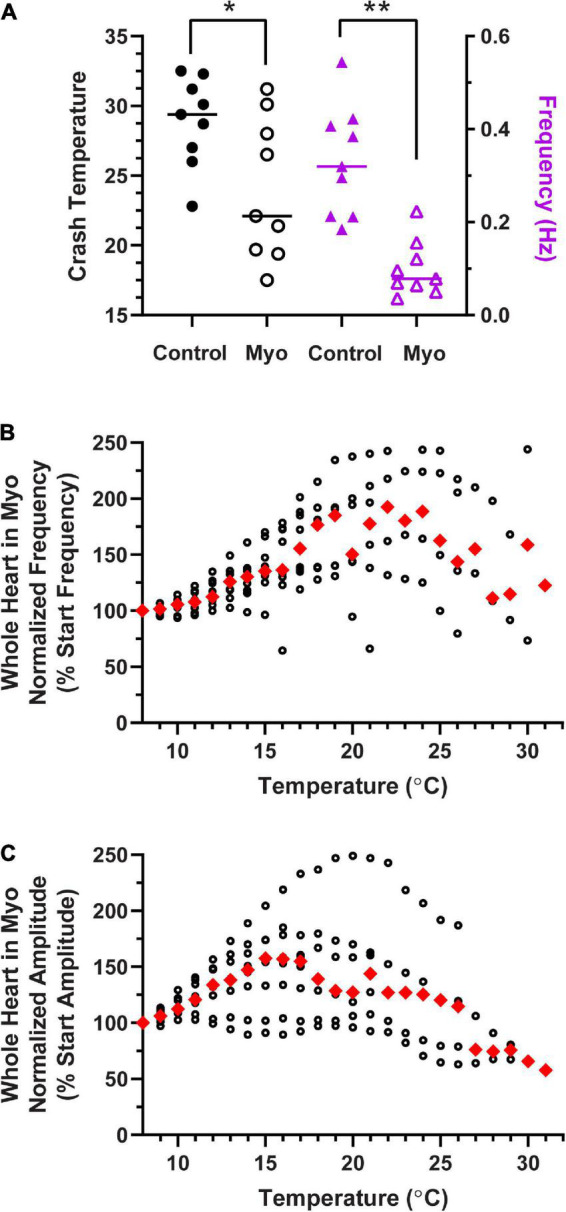
Modulation of the cardiac neuromuscular system by myosuppressin decreases crash temperature. **(A)** Comparison of crash temperatures between preparations perfused with saline (29.4°C, median) vs. 10^– 7^ M myosuppressin (Myo; 22.1°C, median) in the same hearts. Myosuppressin decreased the temperature tolerance of these hearts (Wilcoxon Sign-Rank test: *p* = 0.027, *N* = 9). At the same time, contraction frequency (right y-axis) in all hearts decreased [saline: 0.32 Hz vs. myosuppressin: 0.08 Hz (median frequencies) Wilcoxon test; *p* = 0.004, *N* = 9]. **(B)** Normalized whole heart contraction frequency as a function of temperature during myosuppressin perfusion. Note that in myosuppressin, median contraction frequency initially increased, then decreased with increasing temperature, as is seen in other modulators, but the frequency never dropped below the initial frequency (100%) recorded at 8°C. **(C)** Normalized whole heart contraction amplitude as a function of temperature during myosuppressin perfusion. Contraction amplitude increased with increased temperature until ∼17°C, at which point it decreased, but always remained above 50% of the initial contraction amplitude. Red triangles represent the median response at each temperature step for panels **(B,C)**. *Indicates *p* < 0.05; **Indicates *p* < 0.01.

Similarly, in a separate set of experiments, we perfused the neuromodulator myosuppressin through the whole heart preparation. Figure 12A shows that the crash temperature was significantly decreased by the presence of myosuppressin compared to saline (control) (Wilcoxon Sign-Rank test: *p* = 0.027, *N* = 9). Myosuppressin is a modulator that leads to large decreases in heartbeat frequency, as seen here (Figure 12A, purple triangles; Wilcoxon Sign-Rank test: *p* = 0.004, *N* = 9), and thus generally decreases excitability. Interestingly, when the hearts were exposed to temperature ramps in the presence of myosuppressin, we did not observe a consistent decrease in median normalized contraction frequency until ∼25°C (Figure 12B), and even at that point, median contraction frequency never decreased below the initial frequency (Figure 12B, 100%). In fact, median frequency did not drop below 100% before the preparation crashed. Myosuppressin also typically increases the heartbeat contraction force (Stevens et al., 2009). This was also the case in our experiments when contraction amplitudes were compared at 11°C (mean ± SD 1.12 ± 0.46 g in saline vs. 1.6 ± 0.86 g in Myo; paired *t*-test: *p* = 0.034; Shapiro–Wilk test, *p* = 0.96 for saline and *p* = 0.21 for Myo; *N* = 9), but not at 9°C. In contrast to the patterns recorded in control saline and in other modulators, median normalized contraction amplitude increased with increased temperature until ∼16°C (Figure 12C). At that temperature, it began to decrease. However, the median contraction amplitude never dropped below 50% of the initial median amplitude before crashing in the presence of myosuppressin. While the mechanisms underlying these responses to myosuppressin are not understood, they lay the groundwork for future experiments.

Together, these experiments show that for at least two neuromodulators, when modulation increases neuron excitability, the crash temperature was increased, and when modulation decreased neuron excitability, we conversely observed a decreased crash temperature.

### 3.7. Initial contraction frequency is not a predictor of crash temperature

**FIGURE 13:**
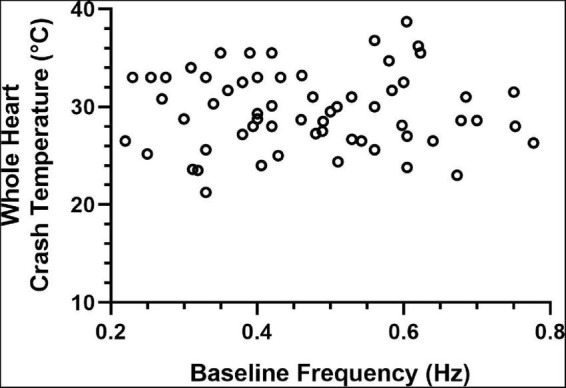
Whole heart crash temperature is not a function of initial contraction frequency. Plot of 63 hearts showing the temperature at which each heart crashed as a function of the baseline contraction frequency of that heart. There is no correlation between the crash temperature in the absence of a modulator (control) compared to the baseline (8–10°C) contraction frequency of each heart (Spearman Rank Correlation test: ρ = 0.069, *d* = 0.54, *p* = 0.59; *N* = 63).

Previous studies have shown that the effects of a modulator may be state-dependent, with the magnitude of the effect dependent on the baseline frequency of the pattern (Nusbaum and Marder, 1989). Because of this, and because some of the data presented here suggest the possibility that the initial contraction frequency may predict or determine the crash temperature of that cardiac neuromuscular system, we examined the relationship between contraction frequency at baseline temperature (8–10°C) and the crash temperature of a group of whole hearts. There was no correlation between baseline frequency and crash temperature, as seen in Figure 13 (Spearman Rank Correlation test: ρ = 0.069, *d* = 0.54, *p* = 0.59; *N* = 63).

## 4. Discussion

### 4.1. Crash temperature changes with burst frequency

All neural circuits are subject to temperature fluctuation and must maintain function across that range. For some nervous systems, that range is homeostatically maintained within a few degrees and for others, that range maps onto temperature fluctuations in the environment, which can be quite large. The dynamics of changes to the whole heart function we observed in our study are similar to those seen previously in the same system (Camacho et al., 2006; Worden et al., 2006; Qadri et al., 2007). Worden et al. (2006) showed that stroke volume (contraction force) decreases with increased temperature and that the contraction frequency increased between 2°C to ∼16–18°C. Consequently, cardiac output did not change significantly between 2–20°C, though it began to decrease at 22°C. Here, we extended the upper range of temperatures tested to enable us to examine the resilience of hearts to increased temperature. As we predicted, based on earlier data from Worden et al. (2006), both contraction frequency and contraction force decreased at higher temperatures as we approached the crash temperature for each heart.

A number of studies have found that signaling molecules and feedback modulation play a role in maintaining circuit function even though the molecular kinetics of all cellular components are altered due to changes in temperature (Zhurov and Brezina, 2005; Hamilton et al., 2007; Biron et al., 2008; Tang et al., 2010; Thuma et al., 2013; Städele et al., 2015; Haddad and Marder, 2018; Takeishi et al., 2020; Städele and Stein, 2022). As such, we were particularly interested in examining the role of feedback in determining the crash temperature in this neuromuscular system. There are two feedback systems that are intrinsic to the lobster cardiac neuromuscular system. (1) Stretching of cardiac muscles during each contraction activates stretch-sensitive dendrites, resulting in a response that has generally been thought to be positive (i.e., increased stretch leads to increased contraction frequency). (2) The muscles contain NO synthase, which releases NO to negatively regulate the heartbeat (i.e., NO slows the frequency, which in turn results in a decrease in stroke volume; Mahadevan et al., 2004). These opposing feedback systems are thought to stabilize the heartbeat.

Because these feedback mechanisms appear to stabilize heart function, we anticipated that the whole heart would be more resilient to increases in temperature compared to the isolated CG, and thus the whole heart would exhibit a higher crash temperature than the isolated CG. However, as we show here, the crash temperature of the isolated CG is in fact significantly higher than that of the semi-intact heart. It is possible that creating the incision in the ventral side of the heart for the semi-intact heart (needed for extracellular recording of the CG, Methods) disrupts the stretch feedback pathway, resulting in minimal positive feedback. However, unpublished data suggest that opening the heart to record neuronal activity does not eliminate responses to stretch. Moreover, this experimental setup would not likely disrupt NO feedback.

Previous studies of temperature tolerance in the lobster heart postulated that the temperature dependence of heart rate arises largely from intrinsic properties of the CG neurons rather than from hormonal or cardio-regulatory neurons (Camacho et al., 2006; Worden et al., 2006). However, a more limited temperature range was used in those studies, and critical temperatures at which the neuromuscular system crashes were not evaluated. Nonetheless, our results are consistent with previous results (Worden et al., 2006), suggesting that there are temperature regulatory mechanisms intrinsic to the CG neurons, though it is likely that the temperature stability of the heart is also affected by cardioregulatory inputs in the intact animal (i.e., modulation). Because the lobster heart is neurogenic, the heartbeat frequency is determined directly by the cycle frequency of the CG. However, it is worth noting that the CG is in turn extensively modulated. Both hormones and cardioregulatory fibers regulate the heartbeat in the intact animal, along with the two aforementioned feedback systems. These feedback systems are left intact in the isolated heart, and hormones can be perfused to assess their impact on cardiac physiology. Thus, we removed all of the modulatory inputs by isolating the CG. Rather than decreasing the temperature tolerance of the CG as predicted, we observed that the isolated CG was more temperature resilient than the semi-intact heart and crashed at a higher temperature. Because these experiments were carried out in the same preparations, we could directly compare the responses from the same CG both in the heart and when isolated from it.

### 4.2. Role of NO in determining crash temperature

The differences in crash temperature of the whole heart vs. the isolated CG led us to ask what role feedback plays in controlling the temperature resilience of the CG. Because we observed lower crash temperatures in the semi-intact heart compared to the isolated nervous systems, it seemed plausible that the relevant difference between the two preparations was the removal of negative feedback processes present in the intact system. NO release presumably occurs continuously, as it is released in response to muscle activity, and the muscles are the only known localization of NO synthase (Mahadevan et al., 2004). Furthermore, because increasing temperature increases the reaction rates of biological processes, NO production likely increases as temperature increases in the semi-intact preparations. It should be noted that in the lobster cardiac nervous system, NO does not activate the cGMP pathway described in vertebrate muscular systems (Goy, 2005). Therefore, while our findings pertaining to the influence of NO on a nervous system’s temperature tolerance may not extrapolate to all NO pathways, it was interesting to find that at least two modulators that decrease neuron excitability also decreased the temperature tolerance of the nervous system.

We examined the role of NO in two ways: (1) applying NO to the isolated CGs and whole hearts using NO donors (PAPA NONOate and SNAP) and (2) blocking NO production in the whole heart using the nitric oxide synthase blocker L-nitroarginine (LNA). Because NO is released from the cardiac muscle in response to cardiac contractions, we would expect it to be present, at least at low levels, in most whole hearts. When we applied NO, using PAPA NONOate, to the whole heart we saw no change in crash temperatures compared to control (saline; Figure 8A). One possible explanation is a ceiling effect, in which concentrations of endogenously produced NO in most preparations were already sufficient such that additional NO did not affect the crash temperature. In contrast, NO was absent in the control saline applied to the isolated CGs (control condition); delivering exogenous NO, using SNAP, to these ganglia decreased the crash temperature in nearly all preparations. Although we applied different NO donors to the whole hearts than to the isolated ganglia, the major difference between these donors is the number of NO molecules released by each. Whereas PAPA NONOate releases two molecules of NO per donor molecule, SNAP donates only one molecule of NO. This means that NO concentrations would likely be higher in the whole hearts than in the isolated CGs.

In a second set of experiments, we blocked NO production in the whole hearts. Because adding NO suggested a ceiling effect of NO presence, we predicted that blocking NO would do two things: (1) increase contraction frequency by removing NO and (2) increase crash temperature if NO is sufficient to decrease crash temperature, as our SNAP data suggest. These were both observed in about half of the whole hearts in which we applied LNA. In those hearts in which LNA did indeed cause an increase in contraction frequency, as predicted based on Mahadevan et al. (2004), crash temperature also increased. Surprisingly, in about half of our hearts, LNA did not cause an increase, but actually resulted in a decreased contraction frequency. Although the mechanism that underlies such a decrease is not clear, it is striking that crash temperature in these preparations did not increase as expected, but instead decreased. These results suggest that positive regulators of excitability extend the temperature range of the heart, and that negative regulators of excitability limit the functional temperature range. This conclusion is corroborated by our experimental results in which exogenous modulators were perfused through the cardiac neuromuscular system.

### 4.3. Temperature robustness may be a function of neuron and circuit excitability

Ours is not the first study to assess whether neuromodulation alters the temperature resiliency of a nervous system (Zhurov and Brezina, 2005; Thuma et al., 2013; Städele et al., 2015; Haddad and Marder, 2018; Powell et al., 2021; Städele and Stein, 2022). Previous data show that activation of the modulator-activated, voltage-sensitive current (I_*MI*_) can extend the temperature range of two other CPGs, the gastric mill and pyloric pattern generators of the stomatogastric system in lobsters and crabs (Städele et al., 2015; Haddad and Marder, 2018; Städele and Stein, 2022). I_*MI*_ voltage sensitivity is similar to that of the NMDA receptor, where membrane depolarization decreases the affinity for a Mg^2+^ ion to the channel pore (Golowasch and Marder, 1992). The I_*MI*_ peak current has been observed between −50 and −10 mV depending on cell type and the modulator used to activate it (Golowasch and Marder, 1992; Swensen and Marder, 2000; DeLong et al., 2009; Rodriguez et al., 2013; Gray and Golowasch, 2016). Importantly, I_*MI*_ currents increase at voltages more depolarized than resting potential, but well within the range of normal oscillations of these neurons, even in the absence of modulator. Because these currents have a negative conductance relationship between resting potential and approximately −10 mV, they can offset the increase in leak current associated with increased temperature (Städele et al., 2015). This may very well be of great importance in the CG, where all synaptic connections directly promote neuron activity (i.e., electrical synapses and excitatory chemical synapses). This is in slight contrast to the STG where intra-STG synapses are inhibitory, and neurons often rely on post-inhibitory rebound for rhythm maintenance. In the pyloric rhythm for instance, increases in leak currents are likely offset to some degree by increases in synaptic conductance (Tang et al., 2010), which may help mitigate some deleterious effects of temperature increase.

Our data indicate that the temperature resilience of a nervous system may have less to do with the specific regulatory pathway influencing it and instead may rely on a more general rule: modulators that upregulate neural excitability increase resiliency and those that decrease excitability, decrease resiliency. This conclusion is supported by our final set of experiments: applying the peptide modulator SGRN, which activates the inward current I_*MI*_, extended the temperature range tolerated by the whole heart, whereas myosuppressin, which decreases neuron excitability (Stevens et al., 2009), decreased the whole heart crash temperature. Although we did not directly assess changes in neuron excitability in response to SGRN, SGRN increases neuron/circuit output at lower concentrations (10^–9^ M; Dickinson et al., 2015) than those used here, which is indicative of increased excitability. While we did not exhaustively apply all regulatory peptides and hormones known to affect the cardiac neuromuscular system, many of the modulators that regulate the cardiac nervous system are thought to act on the same I_*MI*_ conductance (including SGRN) (Swensen and Marder, 2000, 2001). To our knowledge, this is the first set of experiments to examine the relationship between modulators that decrease neuron excitability (NO and myosuppressin) and temperature.

At the present time, the cellular mechanisms that decrease temperature resilience are entirely unknown. There are, however, a few possibilities that seem plausible. Due to the relatively fast response in the heart and isolated ganglion to NO (minutes timescale; Mahadevan et al., 2004), it is likely acting to modify ion channel conductance *via* second messenger signaling (i.e., phosphorylation of channels). Given that NO slows the CG burst frequency but does not alter burst duration, this could include, but is not limited to, upregulating potassium conductances that limit the cycle frequency (e.g., A-type potassium or calcium activated potassium). Prolonged potassium permeability would limit the ability for driver potentials to drive bursting by either decreasing membrane resistance or hyperpolarizing the membrane or both. It could also be the case that there is a down regulation of conductances that tend to decrease time between bursting by leading to slow membrane depolarization [e.g., a persistent sodium current (I_*NaP*_) or the non-selective cation current (I_*CAN*_)]. Although the chloride reversal potential in these neurons is unclear, evidence suggests that E_*Cl*_ may be in the voltage range of neuron activity. If this is the case, NO may be acting on a chloride conductance.

### 4.4. Crash temperature depends on ocean temperature

Even within the same experimental preparation (e.g., isolated CG), crash temperature varied between different experimental groups. For example, the mean crash temperature shown in Figure 6A for those isolated CGs was different than the mean crash temperature for the isolated CGs shown in Figure 8B. Previous work has documented that crash temperature fluctuates with seasonality and ocean temperatures (Marder and Rue, 2021). This is unsurprising as all animals used in these studies are wild caught and their physiology reflects aspects of environmental adaptations beyond our control. In this case, animals caught in colder seasons, when the sea water is colder, typically exhibit a lower crash temperature than animals caught during warmer seasons (Owens, 2014; Marder and Rue, 2021). This is supported by experiments in which animals were maintained for a long period of time at specific acclimation temperatures (Camacho et al., 2006; Tang et al., 2012; Kushinsky et al., 2019; Oellermann et al., 2020). Results of these experiments show that acclimation temperature correlates with crash temperature. This phenomenon indicates that there are likely trade-offs in cell physiology that are temperature dependent. For instance, if it is energetically more expensive to maintain neuronal function in warm water, these processes may arrest during long periods in cold water temperatures.

Data from this study and others (Camacho et al., 2006; Hamilton et al., 2007; Tang et al., 2010; Tang et al., 2012; Thuma et al., 2013; Städele et al., 2015; Kushinsky et al., 2019; Oellermann et al., 2020; Marder and Rue, 2021; Powell et al., 2021; Städele and Stein, 2022) indicate that there are processes endogenous to at least these poikilothermic animals that enable their nervous systems to maintain functional output across a range of temperatures, including those that these organisms are routinely exposed to. However, it would not be surprising to find that these findings generalize across nervous systems. Although this study highlights two such processes (NO and myosuppressin) that decrease temperature robustness of the neuromuscular organ, most of the modulatory processes in the lobster heart act as positive regulators of neural and muscular activity (i.e., other circulating peptides and hormones) (Cruz-Bermudez and Marder, 2007; Christie et al., 2010). Even though it is likely that NO production increases with temperature increase, for the same reasons, it is likely that the production of these modulatory peptides also increases. Furthermore, there may exist evolutionary tradeoffs between the proportion of modulators present in a nervous system that decrease neuron excitability and the temperature tolerance of that system.

## Data availability statement

The raw data supporting the conclusions of this article will be made available by the authors, without undue reservation.

## Ethics statement

Ethical review and approval was not required for the study of animals in accordance with the local legislation and institutional requirements.

## Author contributions

DP, EO, MB, PN, and PD contributed to the conception and design of the study. DP, EO, MB, MC, PN, EB, RJ, and PD conducted experiments and analyzed the data. DP and PD wrote the first draft of the manuscript. All authors contributed to manuscript revision, read, and approved the submitted version.
